# *Palystes kreutzmanni* sp. n. – a new huntsman spider species from fynbos vegetation in Western Cape Province, South Africa (Araneae, Sparassidae, Palystinae

**DOI:** 10.3897/zookeys.67.700

**Published:** 2010-11-10

**Authors:** Peter Jäger, Dirk Kunz

**Affiliations:** Senckenberg Research Institute, Arachnology, Senckenberganlage 25, 60325 Frankfurt am Main, Germany

**Keywords:** Taxonomy, systematics, relationships, species groups, new species

## Abstract

Palystes kreutzmanni **sp. n.** is described from habitats close to Kleinmond, in the Western Cape Province, South Africa. Spiders of this new species live in the typical fynbos vegetation of the Western Cape region. They build retreats between apical leaves of Leucadendron bushes. The systematic position of Palystes kreutzmanni **sp. n.** is discussed. Male and female show characters of different species groups, especially the female copulatory organ seems to be unique within the genus Palystes L. Koch, 1875.

## Introduction

The genus Palystes L. Koch 1875 was revised by Croeser (1996). In that paper he erected a new genus, Parapalystes Croeser, 1996, and transferred to it four species formerly described as Palystes (3 species) and Remmius Simon, 1897 (1 species). He distinguished three species groups within Palystes leaving six species incertae sedis. A cladistic analysis based on 20 morphological characters placed Parapalystes as sister to all Palystes species. Within Palystes, the *castaneus*-group (3 spp.; paraphyletic in Croeser’s analysis) is basal to *lunatus*-group (6 spp.) and *superciliosus*-group (6 spp.). Since that revision Jäger and Kunz (2005) published an illustrated key to the genera of African Sparassidae, including illustrations of 17 Palystinae species. Jäger and Rheims (2010) described the female of Sarotesius melanognathus Pocock, 1898 for the first time and discussed its systematic affiliations. Currently 21 Palystes species are described (Platnick 2010).

During an expedition in South Africa the junior author collected spiders of a species of the subfamily Palystinae Simon, 1897 in Western Cape Province. It is described as a new species and its relationships are discussed. A short identification key is provided as partial update of that of Croeser (1996: 18).

Measurements are in millimetres, arising points of tegular appendages are given as clock-position of the left palp in ventral view. Leg and palp measurements are given as: total (femur, patella, tibia, metatarsus, tarsus). Leg spination is given as: prolateral, dorsal, retrolateral, ventral (the latter digit may be omitted in the case of absence of ventral spines). Female copulatory organs were treated with 96% lactic acid. Material is stored in 70% denatured ethanol.

**Abbreviations:** ALEanterior lateral eyes, AMEanterior median eyes, DKfield numbers of Dirk Kunz, PJsubsequent numbers of Sparassidae examined by Peter Jäger, PLEposterior lateral eyes, PMEposterior median eyes, RTAretrolateral tibial apophysis, SDtissue sample numbers from arachnology collection SMF.

**Museum collections**

ISAMIziko South African Museum, Cape Town (Margie Cochrane)

PPRIPlant Protection Research Institute, Pretoria (Ansie Dippenaar-Schoeman

SMFSenckenberg Research Institute, Frankfurt (Peter Jäger)

## Taxonomy

### Palystes kreutzmanni sp. n.

urn:lsid:zoobank.org:act:D5E6DD79-50F8-4899-AEB3-7674B369A16F

Figs 1 [Fig F2] 25 

#### Type material.

**Holotype male** (PJ 3261), South Africa, Western Cape Province, W of Kleinmond, 34°20'8.69"S, 18°58'32.45"E, 197 m altitude, fynbos vegetation, retreat between apical leaves of Leucadendron sp., by hand, by day, Esther van der Westhuizen and Dirk Kunz leg. 15.V.2004, DK 48, SD 224–225 (SMF). **Paratypes:** **1 female** (PJ 3262), same data as for holotype, except for 34°20'9.708"S, 18°58'32.7"E, 193 m altitude, DK 50, SD 273–274 (SMF). **1 female** (PJ 3266), same data as for preceding specimen except for DK 54, SD 233–234 (ISAM). **1 female** (PJ 3265), same data as for preceding specimen except for DK 52, SD 229–230 (PPRI). **1 female** (PJ 3264), same data as for preceding specimen except for DK 53, SD 231–232 (SMF). **1 female** (PJ 3263), same data as for preceding specimen except for DK 51, SD 2226–227 (SMF). **1 female** (PJ 3267), same data as for preceding specimen except for 34°20'7.7274"S, 18°58'31.9074"E, 192 m altitude, DK 47, SD 228 (SMF).

#### Diagnosis.

**Figures 1–16.**
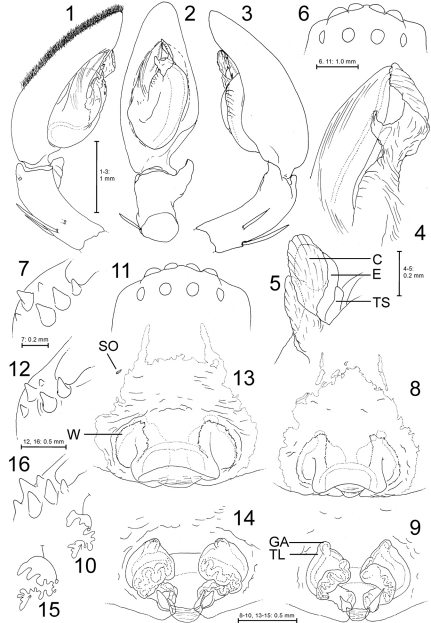
Palystes kreutzmanni sp. n. from South Africa, Western Cape Province, Kleinmond (**1–7** Holotype male, PJ 3261 **8–12** Paratype female, PJ 2362 **13–15** Paratype female, PJ 3267 **16** Paratype female, PJ 3263). **1–5** Palp (1 prolateral, 2 ventral, 3 retrolateral, 4 detail of embolus and conductor, ventral, 5 same, retrolatero-distal). **6, 11** Eye arrangement, dorsal **7, 12, 16** Left chelicera, ventral **8, 13** Epigyne, ventral **9, 14** Vulva, dorsal **10, 15** Schematic course of internal duct system (circle – copulatory orifice, T – glandular appendages, arrow – fertilisation duct in direction of uterus externus). Abbreviations: C – conductor, E – embolus, GA – glandular appendage, SO – slit sense organ, TL – transparent layer, TS – tegular sclerite, W – transparent “window”.

Medium sized Sparassidae, body length of males: 12.7, of females: 13.7–17.0. Males (Figs 1–5) similar to those of Palystes lunatus-group (sensu Croeser 1996), i.e. having a simple and broad embolus and one short and simple RTA, most similar to Palystes leppanae Pocock, 1902. Distinguished by 1. Tegular sclerite (sensu Croeser 1996) situated dorsally of embolus, visible only in retrolatero-distal view, 2. Embolus pointed, straight and almost distad (*lunatus*-group males with retrolaterad embolus), 3. RTA short, stout, with broad massive base and moderately pointed to blunt apex. Females (Figs 8–10, 13–15) may be distinguished from other Palystes species by 1. Posterior margin of median septum distinctly concave as in Palystes stilleri Croeser, 1996, but shorter and not extending to or beyond epigastric furrow, 2. Internal duct system distinctly different from other Palystes spp.: short copulatory ducts leading anteriorly to a wide atrium, where glandular appendages are arising; long wound ducts (= functional spermathecae?) running from medially to lateral, turning medially and then posteriorly to fertilisation ducts, 3. Glandular appendages not free (although it appears as such in a ventral view when observed in lactic acid; Figs 8, 13), but included in a transparent layer covering the anterior part of internal duct system (mainly atrium).

#### Etymology.

In honour of Mr Jürgen Kreutzmann for supporting the systematic research, description of biodiversity and nature conservation in South Africa; noun in genitive case.

#### Description.

##### Male

(holotype, PJ 3261). Prosoma length 6.4, prosoma width 5.1, anterior width of prosoma 2.8, opisthosoma length 6.3, opisthosoma width 3.4. Eyes (Fig. 6): AME 0.39, ALE 0.48, PME 0.35, PLE 0.31, AME–AME 0.15, AME–ALE 0.04, PME–PME 0.39, PME–PLE 0.41, AME–PME 0.45, ALE–PLE 0.29, clypeus height at AME 0.16, clypeus height at ALE 0.21. Spination: Palp: 131, 001, 2121; legs: femur I–III 323, IV 332; patella I–IV 101; tibia 2226; metatarsus I–III 2024, IV 3036. Ventral metatarsus IV distally with median spine embedded in scopula. Leg formula: 2143. Measurements of palp and legs: Palp 7.5 (2.5, 0.9, 1.6, -, 2.5), leg I 29.5 (8.4, 3.5, 8.0, 7.4, 2.2), leg II 30.3 (8.9, 3.5, 8.3, 7.4, 2.2), leg III 23.2 (7.1, 2.8, 6.1, 5.3, 1.9), leg IV 28.0 (8.9, 2.8, 7.2, 7.0, 2.1). Cheliceral furrow with 3 anterior and 3 posterior teeth (Fig. 7).

Palp as in diagnosis. Dorsal scopula covering two apical third of cymbium (Fig. 1). Basal cymbium strongly truncated (Fig. 2). Tibia distinctly shorter than cymbium. Embolus arising in a 8.30-o’clock-position, conductor almost centrally from tegulum. Sperm duct running broad and submarginally at retrolateral tegular margin, narrowing distinctly in S-curve prolaterally and further on its way to subapical opening at embolus. Both margins of embolus slightly convex (Figs 2, 4). Conductor membranous, basally twisted and apically folded.

**Figures 17–21. F2:**
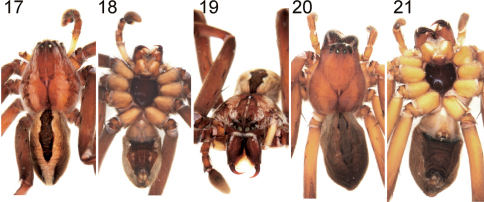
Palystes kreutzmanni sp. n. from South Africa, Western Cape Province, Kleinmond (**17–19** Holotype male, PJ 3261 **20–21** Paratype female, PJ 3263).

**Figures 22–25.**
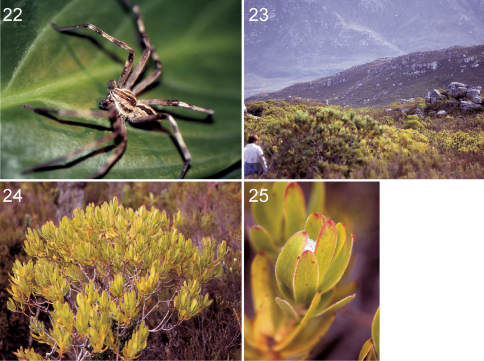
Palystes kreutzmanni sp. n. from South Africa, Western Cape Province, Kleinmond (**22** Holotype male, PJ 3261 **23** Fynbos vegetation at the type locality **24** Leucadendron sp., predominant plant at the type locality **25** Retreat of Palystes kreutzmanni sp. n. between apical leaves of Leucadendron sp.).

Colouration in ethanol (Figs 17–19): Reddish-brown with pattern consisting of white and red hairs and dark markings. Dorsal shield with two lateral longitudinal bands narrowing anteriorly and dark triangular pattern in front of fovea. Indistinct narrow bright median line in front of fovea. Clypeus with transversal band of dense bright hairs. Sternum dark reddish brown, without pattern. Labium dark reddish-brown proximally, with distal bright lip. Gnathocoxae brighter, especially at inner margins. Ventral and retrolateral coxae bright yellowish brown, prolateral sides dark brown. Chelicerae dark reddish brown, with two short longitudinal bands consisting of white hairs, one frontal and one lateral. Palpal femora yellowish brown, rest reddish brown as other appendages. Legs with small spine patches consisting of white hairs, tibiae annulated. Ventral femora I and II with white patch in distal half consisting of white hairs (more distinct in live specimens: Fig. 22). Dorsal tarsi with small longitudinal dark patch in distal half. Dorsal opisthosoma with solid black patch above heart surrounded by bright lanceolate area extending to spinnerets: This brighter area bordered especially in posterior half by darker part. Lateral opisthosoma becoming brighter to ventral side. Ventral opisthosoma with red patch between epigastric furrow and spinnerets becoming blackish anterior and posterior and including one pair of small white patches in the middle and four longitudinal lines of tiny muscle sigillae; further bright dots situated laterally of the red patch. For colouration of live specimen see Fig. 22.

##### Female

(paratype**,** PJ 3262). Prosoma length 6.5, prosoma width 5.2, anterior width of prosoma 3.4, opisthosoma length 8.0, opisthosoma width 5.3. Eyes (Fig. 11): AME 0.39, ALE 0.40, PME 0.31, PLE 0.30, AME–AME 0.16, AME–ALE 0.07, PME–PME 0.41, PME–PLE 0.39, AME–PME 0.40, ALE–PLE 0.37, clypeus height at AME 0.21, clypeus height at ALE 0.25. Spination: Palp: 131, 101, 2121, 1014; legs: femur I–III 323, IV 332; patella I 001, II 0(1)01, III–IV 001; tibia 2126; metatarsus I–III 2024, IV 3036. Ventral metatarsi III–IV with distal median spine embedded in scopula. Leg formula: 2143. Measurements of palp and legs: Palp 7.5 (2.2, 1.1, 1.7, -, 2.5), leg I 24.1 (6.9, 3.2, 6.3, 5.8, 1.9), leg II 24.3 (7.2, 3.2, 6.4, 5.7, 1.8), leg III 18.3 (5.7, 2.5, 4.6, 4.1, 1.4), leg IV 22.6 (7.2, 2.5, 5.7, 5.5, 1.7). Cheliceral furrow with 3 anterior and 2 posterior teeth (Fig. 12, but see “Variation”, Fig. 16). Palpal claw with 6 teeth.

Copulatory organ as in diagnosis. Epigynal field as long as wide with narrow anterior bands. No slit sense organs present close to epigynal field (but see “Variation”, Fig. 13). Bright transparent areas (“windows”) situated anterolaterally of median septum. Median septum containing a large blunt cavity, most likely for accommodating the male RTA during copulation process (Figs 8, 13; as observed, e.g., in Heteropoda spp., Jäger unpubl.). Internal duct system with additional external layer (Fig. 9: TL) concealing the original profile of the ducts. Jäger (2008: 283, figs 268, 271) reported on this phenomenon in Heteropoda homstu Jäger, 2008, considering evidences from subadult and adult conspecifics. Fertilizations ducts relatively large, pointing anterio-laterally.

Colouration in ethanol (Figs 20–21): As in male but in general with less distinct pattern. Pattern of dorsal shield of prosoma barely recognisable, heart patch of dorsal opisthosoma indistinct. Legs with fewer and shorter white hairs. Prolateral coxae bright yellowish brown. Chelicerae without lateral bright longitudinal band.

#### Variation.

Females (n=6): Spination: femur III 323(2), patellae I 101/001, II–IV 101/001/000. Chelicerae with 3 posterior teeth (Fig. 16).

Colouration: in general there were distinct differences in contrast and strength of the pattern. Dorsal opisthosoma with patch above heart only marginally black, uniformly grey or with same colour as surrounding areas; in one female (PJ 3264) one lateral pair of small white patches was included in the middle of the heart. Ventral opisthosoma with paired white patches and white dots varying in size, shape and position. In one female (PJ 3266) an additional small unpaired white median patch was present between the paired patches and the spinnerets. Pattern of prolateral coxae varying from entirely yellowish brown to having dark patches to a different degree with stronger markings in anterior coxae. Frontal longitudinal white bands of chelicerae varying in length.

Copulatory organ: In one female (PJ 3267) one slit sense organ was present (Fig. 13). Internal duct system may be more compact and right and left half more separated (Fig. 14). The median septum may be broader (Fig. 13) or exhibiting a median bulge in the concave posterior margin.

#### Distribution.

Only known from the type locality (Figs 23–24).

#### Biology.

Retreats were built between apical leaves of Leucadendron plants (Fig. 25). Spiders were resting here during the day time.

#### Relationships.

Palystes kreutzmanni sp. n. cannot be assigned to any of the species groups listed in Croeser (1996), as it shows a combination of character states conflicting with one or the other diagnosis including that of the genus Parapalystes. According to Croeser (1996) the colouration of the new species shows affinities to the *castaneus*-group (solid black sternum), *superciliosus*-group (ventral femora I–II with a bright patch apically), and to Parapalystes (solid black sternum, heart patch solid brown in males). The female epigyne exhibits a uniquely shaped median septum (not tongue-like as in *lunatus*-group). It is most similar to Palystes stilleri, but shape and course of the internal duct system is distinctly different than in any other species group listed above. There seems to be a general bauplan of the internal duct system recognisable for Palystinae (incl. Palystes, Parapalystes, Sarotesius Pocock, 1898, Panaretella Lawrence, 1937, Anchonastus Simon 1898, ?Staianus Simon 1889): copulatory opening followed by an atrium (may be elongated), glandular appendages arising from this atrium, subsequent (in many species wound) ducts leading to fertilisations ducts. A median septum together with adjacent furrows is present, only in Anchonastus plumosus (Pocock, 1899) furrows in the anterior part are fused and not recognisable at the cuticular surface. Palystes kreutzmanni sp. n. is unique in having the first intromittent part running from lateral antero-medially (in Palystes superciliosus group starting from median running antero-laterally).

Number of tibial spines should differentiate between two genera (Croeser 1996: 86; 1 in Parapalystes, 2 in Palystes). In Palystes kreutzmanni sp. n., however, males have two spines (n=1), females have one spine (n=6; a sexual dimorphism known from many Heteropodinae, e.g., Heteropoda davidbowie Jäger, 2008, or from the genus Rhitymna Simon 1897: Jäger 2003, 2008).

Considering all these observations Palystes kreutzmanni sp. n. may be one species with mixed apomorphic character states, or characters previously used for differentiating species groups within Palystinae were symplesiomorphic. However, in both cases only a thorough revision of all taxa in question can help to understand character evolution and phylogenetic position of species and genera, and finally placing the new species correctly.

In the identification key of Croeser (1996) **males** key out (if considering sternum of the new species black) at #16. Here a trichotomy should be inserted:

**Table:** 

16(15)	Tibial apophysis three-lobed; embolus straight; conductor elongate, straight (Cape Town, Stellenbosch, Somerset West and Bredashorp districts, western Cape, South Africa)	Palystes castaneus
–	Tibial apophysis entire; embolus recurved through 90° over bulb; conductor short, bowl shaped	17
	(leading to Palystes martinfilmeri and Palystes stilleri)	
–	Tibial apophysis entire; embolus short, straight, pointed; conductor short, slightly bowl-shaped (western Cape, South Africa)	Palystes kreutzmanni sp. n.

**Females** key out at #7. At this point a trichotomy should be inserted:

**Table:** 

7(2)	Sternum entirely black; femora I–II mottled without distinct markings; opisthosoma laterally mottled, without distinct markings, ventrally with black bell-shaped mark or black-framed yellow panel between black crescent and spinnerets; septum posteriorly produced laterally (western Cape, South Africa)	8
	(leading to Palystes stilleri, Palystes martinfilmeri and Palystes castaneus)	
–	Sternum usually with two mesally interrupted transverse dark bars (sometimes faint, sometimes with an additional short longitudinal bar mesally); femora I–II ventrally with clear white spots; opisthosoma laterally with clear white spots, ventrally rich red to orange-red with distinct clear white spots between black crescent and spinnerets (indistinct in specimens from Amatola Mountains, eastern Cape, South Africa); septum with transverse or posteriorly produced median lobe	**10**
	(leading to Palystes crawshayi, Palystes stuarti, Palystes lunatus, Palystes perornatus, Palystes leppanae and Palystes karooensis)	
–	Sternum dark reddish brown to black without transverse bars; femora I–II ventrally with one single white distal patch ventrally and bright spine patches (indistinct in ethanol); opisthosoma laterally with indistinct irregular pattern, ventrally dark red to black with indistinct white spots mostly in anterior half; septum posteriorly produced laterally, copulatory ducts with cover of sclerotised layer concealing glandular appendages (western Cape, South Africa)	Palystes kreutzmanni**sp. n.**

## Supplementary Material

XML Treatment for Palystes kreutzmanni

## References

[B1] Croeser PMC (1996) A revision of the African huntsman spider genus *Palystes* L. Koch, 1875 (Araneae: Heteropodidae). Annals of the Natal Museum 37:1-122

[B2] Jäger P (2003)*Rhitymna* Simon 1897: an Asian, not an African spider genus. Generic limits and description of new species (Arachnida, Araneae, Sparassidae). Senckenbergiana biologica 82 (1/2): 99-125

[B3] Jäger P (2008) Revision of the huntsman spider genus *Heteropoda* Latreille 1804: species with exceptional male palpal conformations (Araneae: Sparassidae: Heteropodinae). Senckenbergiana biologica 88 (2):239-310

[B4] Jäger P Kunz D (2005) An illustrated key to genera of African huntsman spiders (Arachnida, Araneae, Sparassidae). Senckenbergiana biologica 85 (2):163-213

[B5] Jäger P Rheims CA (2010) First description of the female of *Sarotesius melanognathus* Pocock 1898 (Araneae: Sparassidae: Palystinae). Journal of Arachnology 38 (2):368-370

[B6] Platnick NI (2010)*The world spider catalog, version 11.0*. American Museum of Natural History Available from: http://research.amnh.org/iz/spiders/catalog/INTRO1.html[accessed 24.IX.2010].

